# Asthma bronchiale und allergische Rhinitis – die Hautprobe offenbart eine schwerwiegende Systemerkrankung

**DOI:** 10.1007/s00105-024-05323-w

**Published:** 2024-03-19

**Authors:** Priscila Wölbing, Susanne Dugas-Breit, Wolfgang Hartschuh, Ferdinand Toberer

**Affiliations:** 1 Hautklinik, Universitätsklinikum Heidelberg, Im Neuenheimer Feld 440, 69120 Heidelberg, Deutschland; 2 Drs. Durani, Haut- und Laserzentrum Heidelberg, Heidelberg, Deutschland

**Keywords:** Asthma bronchiale, Eosinophile granulomatöse Polyangiitis, Eosinophilie, ANCA-assoziierte Vaskulitiden, Churg-Strauss-Syndrom, Late onset Asthma bronchiale, Late-onset asthma bronchiale, Eosinophilic granulomatosis with polyangiitis, Eosinophilia, ANCA-associated vasculitis, Churg-Strauss syndrome, Late onset Asthma bronchiale

## Abstract

Vorgestellt wird eine 30-jährige Patientin, die seit Jahren an initial unspezifischen Symptomen, wie rezidivierenden, nicht allergischen und nicht infektiösen Sinusitiden, „late onset“ Asthma bronchiale und ausgeprägter Lymphadenopathie litt. Erst nach Auftreten erster Hauterscheinungen konnte durch die Entnahme einer Probebiopsie die Diagnose einer eosinophilen granulomatösen Polyangiitis (EGPA) gestellt werden. Die EGPA ist eine schwerwiegende Systemerkrankung, die unbehandelt multiple Organschäden verursachen und sogar einen tödlichen Verlauf nehmen kann. Mit der adäquaten Behandlung verläuft die Erkrankung in mehr als 90 % der Fälle milde und kann oft sogar vollständig in Remission gehen. Durch die richtige Diagnosestellung konnte die Patientin adäquat und erfolgreich behandelt und das Risiko für Spätmanifestationen und Folgeschäden mit potenziell tödlichem Verlauf vermindert werden.

## Abstract (en)

This article reports the case of a 30-year-old female patient who suffered for many years from initially unspecific symptoms, such as recurrent, nonallergic and noninfectious sinusitis, late-onset bronchial asthma and pronounced lymphadenopathy; however, the correct diagnosis of eosinophilic granulomatosis with polyangiitis (EGPA) could only be made by histological investigations after the appearance of skin symptoms. The EGPA is a severe systemic disease which, if left untreated, can cause multiple organ damage and even be fatal. With adequate treatment the disease is mild in more than 90% of cases and patients can even completely recover. By making the correct diagnosis, the patient could be successfully treated and the risk of late manifestations and subsequent damage with a potentially fatal outcome was reduced.

## Anamnese

Eine 30-jährige Patientin stellte sich 2023 in der dermatologischen Ambulanz mit seit 5 Monaten bestehenden, flächigen Hautveränderungen der Unterschenkel prätibial beidseits sowie am Kapillitium hochfrontal rechts vor. Ähnliche Erscheinungen seien bisher nicht aufgetreten, aber die Patientin berichtete über ein Asthma bronchiale sowie eine allergische Rhinitis und Sinusitis, seit ca. 2016 bestehend. Nebenbefundlich zeigte sich eine seit 2018 bestehende schmerzlose Lymphadenopathie zervikal und axillär beidseits, die histologisch und bildmorphologisch als reaktiv und unspezifisch eingeschätzt wurde. Die Patientin gab außerdem ein subjektives Gefühl von Abgeschlagenheit und Kraftminderung in den letzten 6 Monaten sowie Kribbelparästhesien palmar und plantar an.

## Untersuchung

**Abb. 1:**
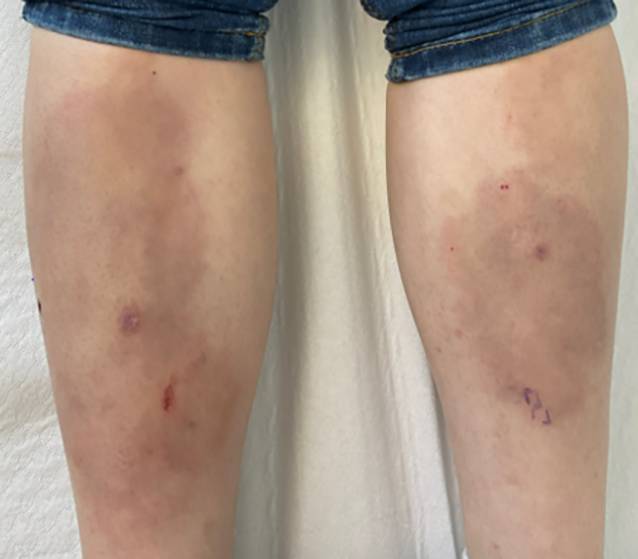
An den Unterschenkeln beidseits imponieren randbetonte, hyperpigmentierte, gelblich-bräunliche, großflächige Plaques

Bei Vorstellung zeigten sich an den Unterschenkeln prätibial beidseits flächige, zum Teil unscharf begrenzte, randbetonte, livid-hyperpigmentierte Makulae bis Plaques mit zentraler Hypopigmentierung (Abb. 1). Am Kapillitium hochfrontal rechts tastete sich ein derber, ca. 1 cm durchmessender, verschieblicher, indurierter Knoten. Zudem bestanden weiterhin prall elastisch zu tastende, verschiebliche, nicht druckdolente Lymphknoten submandibulär, zervikal und axillär beidseits.

## Diagnostik

**Abb. 2:**
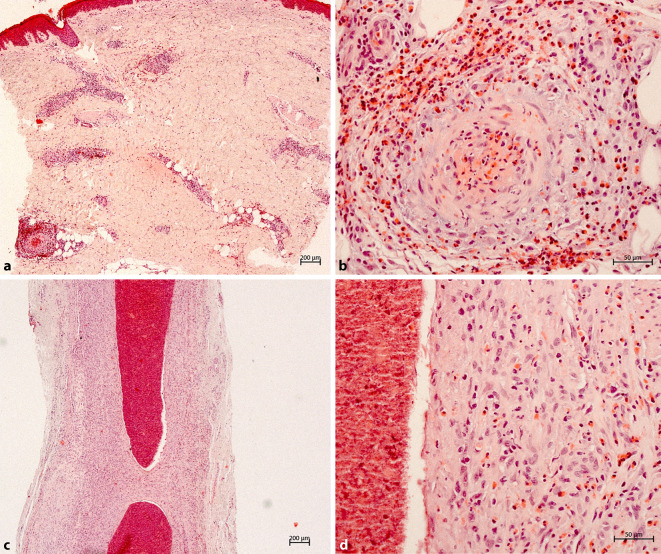
**a** Exzidat aus dem Randbereich der Plaque am linken Unterschenkel. **b** Ausschnittvergrößerung. **c** Exzidat von der Schläfe rechts mit Anschnitt einer Arterie. **d** Ausschnittvergrößerung der Gefäßwand

In der vom linken Unterschenkel aus dem Randbereich entnommenen Probebiopsie (Abb. 2a, b) zeigte sich histologisch eine Orthokeratose mit unauffälliger Epidermis. Auffällig imponierte ein dermal bis subkutan gelegenes, perivaskuläres Infiltrat aus massenhaft eosinophilen Granulozyten, diese waren auch diskret interstitiell nachweisbar. Zusätzlich bestand das Infiltrat aus Lymphozyten und Histiozyten. Ein ebensolches Infiltrat zeigte sich auch um die Schweißdrüsen. Zum Teil fand sich in einem mittelgroßen kutanen Gefäß eine fibrinoide Verquellung der Endothelien mit Durchsetzung der Gefäßwände durch eosinophile Granulozyten. Ferner zeigten sich keine Erythrozytenextravasate und nur eine diskrete Leukozytoklasie.

In der Probebiopsie vom Kapillitium hochfrontal rechts (Abb. 2c, d) zeigte sich ein Anschnitt eines großen Gefäßes, dessen Wand komplett durchsetzt waren mit einem dichten Infiltrat aus reichlich eosinophilen Granulozyten mit Leukozytoklasie, vereinzelt Lymphozyten und Histiozyten. Das Gefäßlumen war mit Thromben verlegt. In der Elastica-van-Gieson-Färbung Darstellung einer kräftigen Membrana elastica interna, passend zu einer Arterie.

Somit ergab sich histologisch der Verdacht auf eine eosinophile Granulomatose mit Polyangiitis (EGPA). In der direkten Immunfluoreszenzuntersuchung zeigte sich C3c granulär perivaskulär, vereinbar mit einer Vaskulitis. In der laborchemischen Untersuchung fanden sich erhöhte Werte für eosinophile Granulozyten bis zu 5,26/nl (40,4 %), Normbereich < 0,5/nl (2–4 %). Darüber hinaus zeigten sich ein erhöhter CRP(C-reaktives Protein)-Wert von 48,3 mg/l (< 5 mg/l) sowie eine erhöhte Blutsenkungsgeschwindigkeit von 26 mm/h (< 20 mm/h). Das Gesamt-IgE war ebenfalls mit 3815 U/ml (< 100 U/ml) deutlich erhöht. Auch der ANA-Titer war mit 1:80 (< 1:80) grenzwertig erhöht, ANCA zeigten sich in der indirekten Immunfluoreszenzuntersuchung negativ.

Aufgrund der asthmatischen Beschwerdesymptomatik unter Berücksichtigung des Hautbefundes und der Histologie wurde bei Verdacht auf eine EGPA eine CT-Untersuchung des Thorax veranlasst. Diese ergab multiple konsolidierende pulmonale Milchglasinfiltrate, vereinbar mit eosinophilen Infiltraten.

In der Lungenfunktionsprüfung zeigte sich eine leichtgradig eingeschränkte CO-Diffusionskapazität sowie ein leichtgradig eingeschränkter CO-Transferkoeffizient mit auch erniedrigtem korrigiertem O_2_-Partialdruck von 77 mm Hg in der kapillären Blutgasanalyse.

Nebenbefundlich fand sich am rechten Unterschenkel lateral, parallel zur Achillessehne verlaufend, ein derb palpabler, nicht dolenter Strang, der sich sonographisch als thrombosiertes Blutgefäß im Rahmen der Grunderkrankung beziehungsweise Thrombophlebitis darstellte.

In Gesamtschau der genannten Befunde konnte die Diagnose einer EGPA gestellt werden.

## Therapie und Verlauf

Die Patientin wurde in unserer rheumatologischen Abteilung zur interdisziplinären Behandlung vorgestellt. Gemeinsam wurde bei Vorliegen einer mittelschweren Erkrankung, guten Erfahrungswerten und guter allgemeiner Verträglichkeit sowie unter Berücksichtigung des Patientenwunsches initial die Entscheidung über die Einleitung einer Prednisolon-Stoßtherapie mit 60 mg täglich über 3 Tage und stufenweiser Reduktion bis zur Erhaltung mit Prednisolon 2,5 mg täglich getroffen. Additiv wurde bei ausgeprägtem pulmonalem Befund eine Basistherapie mit Azathioprin 50 mg täglich eingeleitet. Im Verlauf wurde die Dosierung von Azathioprin auf 100 mg täglich gesteigert. Darunter zeigten sich die Hautläsionen langsam regredient bei ansonsten residuellen postinflammatorischen Hyperpigmentierungen an den Unterschenkeln prätibial beidseits. Das Allgemeinbefinden der Patientin besserte sich rasch.

## Diskussion

Die EGPA, früher auch unter dem Synonym Churg-Strauss-Syndrom, nach den Erstbeschreibern, bekannt, ist eine schwerwiegende Systemerkrankung, die entsprechend der „2012 Revised International Chapel Hill Consensus Conference Nomenclature of Vasculitides“ zu den ANCA-assoziierten Vaskulitiden gezählt wird [1], wobei nur ungefähr 30 % der Fälle auch tatsächlich ANCA-positiv sind. Die EGPA ist mit einer Inzidenz von 0,5–4,2 Fällen je eine Million Einwohner jährlich eine seltene Erkrankung. Die Prävalenz liegt zwischen 10 und 14 Fällen pro eine Million Einwohner. Das Geschlecht scheint dabei kein Einflussfaktor zu sein, vielmehr sind Männer und Frauen gleichermaßen betroffen. Der Erkrankungsdurchschnitt liegt bei etwa 50 Jahren. Kinder sind sehr selten betroffen [2].

Die Differenzierung zu anderen ANCA-assoziierten Vaskulitiden oder Erkrankungen, die mit einer Eosinophilie einhergehen, gestaltet sich aufgrund der initial oft unspezifischen Symptome schwierig. Dabei sind gerade bei der EGPA die korrekte Diagnosefindung und adäquate Behandlung unabdingbar, um Folgeschäden und potenziell tödliche Verläufe zu verhindern. Behandelt ist die Prognose exzellent und die Mortalität sehr gering [3].

Die EGPA präsentiert sich für gewöhnlich in 3 klinischen Phasen [4]. Initial treten meist eine allergische Rhinitis und Sinusitis sowie in 95–100 % der Fälle asthmatische Beschwerden auf. Die initial sehr unspezifischen Beschwerden, die in der Allgemeinbevölkerung häufig sind und auch bei anderen Erkrankungen, etwa bei Pollenallergien, auftreten können, sind in dieser Phase wenig hilfreich für die Diagnosefindung. Gerade bei einem spät auftretenden Asthma bronchiale sollte man daher immer auch an die Möglichkeit einer EGPA denken. Vor allem für inhalative Allergene, die zu allergisch bedingten, asthmatischen Beschwerden führen, wie bei einer Allergie gegen Hausstaubmilben oder Pollen, zeigen sich Allergietests meist negativ. Weiterhin ist eine Sputumeosinophilie ein wichtiges Kriterium zur Diagnosefindung. Die erste Phase verläuft in der Regel über eine Latenzzeit von 8–10 Jahren. Die folgende Phase ist geprägt durch einen Anstieg der eosinophilen Granulozyten. Klinisch präsentiert sie sich mit Entzündungssymptomen verschiedener Organsysteme, am häufigsten sind Magendarmtrakt, Herz oder Lunge betroffen. In der bildgebenden Untersuchung der Lunge finden sich typischerweise flüchtige Infiltrate. Eine ausgeprägte kardiale Entzündung mit Infiltration eosinophiler Granulozyten und einer dadurch bedingten Kardiomyopathie kann zu kardialem Versagen führen und stellt die häufigste Todesursache bei der EGPA dar [5]. Typischerweise besteht eine Bluteosinophilie von meist > 10 %, wobei die Zahl der eosinophilen Granulozyten mit der Krankheitsaktivität korreliert [6]. Oft sind Entzündungsparameter wie CRP und Blutsenkungsgeschwindigkeit erhöht. Es können diverse weitere klinische Manifestationen bestehen, fast immer kommt es zu unspezifischen Allgemeinsymptomen, wie Abgeschlagenheit und Fieber, aber auch zu Arthralgien sowie zu Symptomen einer Mono- oder Polyneuropathie. Die zweite Erkrankungsphase kann ebenso wie die erste Phase der Erkrankung mit einer längeren Latenz von mehreren Jahren auftreten. Gerade in dieser Phase ist die Abgrenzung zum Hypereosinophiliesyndrom oft schwierig [7].

Die dritte Phase der EGPA ist gekennzeichnet durch eine generalisierte Entzündung der Gefäße, wobei verschiedene Gefäße in verschiedenen Organen betroffen sein können. In ca. 40 % der Fälle kommt es zu einer Hautbeteiligung mit palpabler Purpura, Petechien, subkutanen Knoten und Papeln und einer histologisch nachweisbaren perivaskulären Entzündung der dermalen Gefäße. Eine renale Beteiligung steht dabei, im Gegensatz zu anderen Vaskulitiden, nicht im Vordergrund, kommt aber in 20–30 % der Fälle vor und verläuft in ca. 5 % der Fälle dann auch schwer als „rapid progressive glomerulonephritis“ [8].

Die Granulomatose mit Polyangiitis und die mikroskopische Polyangiitis, die ebenso zu den ANCA-assoziierten Vaskulitiden gezählt werden, stellen wichtige und schwierig abzugrenzende Differenzialdiagnosen dar. Die Granulomatose mit Polyangiitis präsentiert sich dabei aber ohne asthmatische Beschwerden. Bei der mikroskopischen Polyangiitis besteht dagegen keine relevante Blut- und Gewebeeosinophilie.

Interessanterweise stehen bei den ANCA-assoziierten Formen der EGPA vor allem die klinischen Symptome einer Vaskulitis im Vordergrund. Patienten mit ANCA-positiver EGPA entwickeln häufiger Symptome, wie sie auch bei anderen ANCA-assoziierten Vaskulitiden auftreten: Dies sind vor allem Neuropathien, Hautmanifestationen und Glomerulonephritiden. Bei den ANCA-negativen Formen stehen die klinischen Symptome einer Eosinophilie eher im Vordergrund. Dies sind insbesondere allergische Rhinitis, Polyposis und Sinusitis, Asthma und kardiale Symptome. Die Übergänge sind fließend, und häufig bestehen Mischformen beider Symptomkomplexe.

Als diagnostische Hilfskriterien wurden vom American College of Rheumatology folgende Kriterien aufgelistet:

- Asthma bronchiale,
- Eosinophilie (> 10 %) des Blutes,
- Mono- oder Polyneuropathie,
- radiologisch nachweisbare wandernde pulmonale Infiltrate,
- akute oder chronisch rezidivierende Sinusitiden und
- bioptischer Nachweis einer extravaskulären Eosinophilie.

Das Zutreffen von mindestens 4 dieser Kriterien macht die Diagnose einer EGPA wahrscheinlich [9].

Ursächlich auslösende Faktoren sind nicht bekannt. Diskutiert werden Umweltfaktoren und inhalative Noxen sowie auch eine Triggerung durch Medikamente oder Vakzinierungen. Es scheint eine T‑Zell-vermittelte Immunreaktion mit vermehrter IL(Interleukin)-5-Ausschüttung und dadurch vermehrter Aktivierung der eosinophilen Granulozyten zu bestehen. IL‑5 spielt bei Ausreifung, Migration und Proliferation der eosinophilen Granulozyten eine entscheidende Rolle. Entsprechend gibt es bereits einige erfolgversprechende Therapieansätze mit den Anti-IL-5-Antikörpern Mepolizumab und Benralizumab [10]. Aktuell empfiehlt die Leitlinie zu Diagnostik und Management der EGPA von Juni 2023 eine Initialtherapie der EGPA mit hochdosierter Glukokortikoiden alleine zur Remissionsinduktion bei leichten Fällen. In leichten Fällen konnten außerdem gute Ergebnisse bei der Gabe von Glukokortikoiden in Kombination mit dem monoklonalen IL-5-Antikörper Mepolizumab gezeigt werden [2]. In schweren Fällen wird die Hinzunahme von Cyclophosphamid oder Rituximab empfohlen. Zur weiteren Therapie wird bei leichten Fällen zur Remissionserhaltung eine niedrigdosierte Glukokortikoidtherapie, ggf. in Kombination mit Mepolizumab empfohlen. In schweren Fällen zeigte sich zur Remissionserhaltung eine Therapie mit Rituximab als gut wirksam.

## Fazit für die Praxis

- Die EGPA (eosinophile granulomatöse Polyangiitis) ist eine schwerwiegende Systemerkrankung, die unbehandelt multiple Organschäden verursachen und sogar einen tödlichen Verlauf nehmen kann. Als häufigste Todesursache steht ein kardiales Versagen im Vordergrund.
- Mit der adäquaten Behandlung verlaufen mehr als 90 % der Erkrankungen milde und können oft sogar vollständig in Remission gehen. Allerdings tritt bei mehr als 40 % der Patienten ein Rezidiv auf.
- Unbehandelt besteht vor allem in der vaskulitischen Phase eine hohe Mortalität innerhalb der ersten 3 Monate.
- Die Diagnose zu stellen sowie rechtzeitig und adäquat zu behandeln, ist von enormer Wichtigkeit, um Folgeschäden und potenziell tödliche Verläufe zu verhindern sowie um Rezidive frühzeitig wieder „einzufangen“.
